# 自体造血干细胞移植序贯CD19 CAR-T细胞治疗完全缓解的B淋巴母细胞淋巴瘤2例

**DOI:** 10.3760/cma.j.cn121090-20251202-00565

**Published:** 2026-05

**Authors:** 晓宁 王, 娟 任, 迪 吴, 鹏程 贺

**Affiliations:** 西安交通大学第一附属医院血液内科，西安 710061 Department of Hematology, the First Affiliated Hospital of Xi'an Jiaotong University, Xi'an 710061, China

例1，男，29岁，2025年2月因“发现左侧锁骨上肿物2周余”就诊于我院，查血常规：WBC 3.84×10^9^/L、HGB 159 g/L、PLT 271×10^9^/L；行局部肿物活检，病理提示B淋巴母细胞淋巴瘤/白血病，免疫组化：CD20（−）、CD3（T细胞，+）、CD5（T细胞，+）、CD10（灶+），Bcl-6（−）、CD21（−）、Ki-67（70％+）、CD79a（弥漫+）、MUM1（−）、Bcl-2（弥漫+）、CylinD1（−）、CD138（−）、CD23（−）、TdT（弥漫+）、CD34（弥漫+）、P53（野生型）、PAX-5（弥漫+）、CD45RO（散在+），骨髓提示增生性骨髓象。骨髓流式细胞术检测未见免疫表型异常细胞。PET-CT提示：左侧锁骨区软组织肿块，左侧胸大肌、胸骨前片絮状密度增高影，考虑淋巴瘤可能；左侧竖脊肌、左侧锁骨^18^F-氟代脱氧葡萄糖（FDG）代谢增高，考虑淋巴瘤组织不除外。LDH水平正常。染色体核型46, XY。诊断：B淋巴母细胞淋巴瘤/白血病（标危组）。

患者接受VILP方案（长春瑞滨40 mg，第1、8、15、22天；伊达比星10 mg第1～3天，5 mg第4天，10 mg第15～17天；培门冬酶3 750 U第22天；泼尼松60 mg第1～28天）及CAML方案（环磷酰胺1.8 g，第1天；阿糖胞苷140 mg，第3～6天、第13～16天；巯嘌呤90 mg，第1～14天）后复查PET-CT提示：原左侧锁骨区软组织肿块及左侧胸大肌、胸骨前片絮状密度影此次未显示，左侧锁骨未见^18^F-FDG代谢增高灶，多维尔评分1分，考虑完全代谢缓解。鉴于患者为青年男性，完全缓解但存在Ki-67（70％+），且无非血缘HLA相合供者以及血缘供者行allo-HSCT，经医院伦理审批（XJTU1AF2025LSYY-922）及患者充分知情同意后，选择探索性进行auto-HSCT序贯嵌合抗原受体T（CAR-T）细胞治疗。

2025年6月给予G-CSF联合普乐沙福稳态动员后采集自体外周血造血干细胞及淋巴细胞，采用靶向CD19的二代CAR结构（含4-1 BB共刺激域）的慢病毒载体转导制备CAR-T细胞。6月10日采集单个核细胞7.92×10^8^/kg，CD34阳性细胞计数4.18×10^6^/kg。6月11日采集单个核细胞2.97×10^8^/kg，CD34阳性细胞计数1.2×10^6^/kg。其后给予奥加伊妥珠单抗1 mg治疗2次，整个治疗周期内行鞘内注射3次。采用改良的MEAM方案［米托蒽醌脂质体24 mg/m^2^，移植前第7天（−7 d）；阿糖胞苷200 mg/m^2^，间隔12 h 1次，−6～−3 d；依托泊苷200 mg/m^2^，−6～−3 d；美法仑140 mg/m^2^，−2 d］进行预处理，2025年7月8日（0 d）回输6月10日采集的自体外周血造血干细胞。CAR-T细胞产品（纳基奥仑赛注射液，含CAR-T细胞计数0.6×10^8^），放行前进行质量控制，检测结果合格，可供临床使用。2025年7月14日（+6 d）回输自体CD19-CAR-T细胞，同时使用左乙拉西坦预防癫痫。密切监测移植后植入情况、CAR-T细胞治疗相关不良事件。在CAR-T输注后第7天、14天、28天、3个月监测CAR-T细胞扩增情况，并采用PET-CT或增强CT进行疗效评估。CAR-T细胞回输后第14天达扩增高峰，之后逐渐下降，扩增的CAR-T细胞主要为CD8^+^ CAR-T细胞。患者造血尚未重建，+20 d再次回输6月11日采集的外周血造血干细胞；+30 d粒系重建，+35 d血小板重建；在CAR-T细胞输注后第2天出现发热，体温最高37.8 °C，第3天最高体温38.3 °C，给予对乙酰氨基酚对症治疗后发热仍反复，后续最高升至40.3 °C，血压及血氧饱和度正常，IL-6水平变化不明显，诊断1级细胞因子释放综合征（CRS），给予地塞米松5 mg/d治疗后体温正常，随访至2025年12月1日，患者处于可检测残留病（MRD）阴性缓解状态。

例2，男，33岁，2025年5月因“持续性胸痛不适1周”就诊于我院，查血常规：WBC 8.1×10^9^/L，HGB 180 g/L，PLT 289×10^9^/L，胸部增强CT提示：左侧后纵隔（第7至第9胸椎水平）病变，轻度强化，结核感染T淋巴细胞检测阳性，给予利福平、吡嗪酰胺等治疗2周，期间仍胸痛。进一步行气管镜检查及超声引导下左侧胸膜组织活检，术后病理提示B淋巴母细胞淋巴瘤/白血病，免疫组化：TDT（+）、CD20（−）、CD3（−）、CD7（−）、CD117（+）、PAX5（部分+）、P53（50％+）、Ki-67（70％+）。骨髓涂片提示增生性骨髓象。骨髓流式未见免疫表型异常细胞。2025年5月14日PET-CT提示：左侧后纵隔软组织影，^18^F-FDG代谢增高，符合B淋巴母细胞淋巴瘤/白血病表现。LDH水平正常。染色体核型46,XY。诊断：B淋巴母细胞淋巴瘤/白血病（标危组）。

患者接受VIP方案（长春瑞滨40 mg，第1、8、15、22天；伊达比星10 mg，第1～3天、第15～17天；泼尼松50 mg，第1～28天）以及CAML方案（环磷酰胺2 g，第1天；阿糖胞苷150 mg，第3～6天、第10～13天；培门冬酶3 750 U，第2天；巯嘌呤100 mg，第1～13天）化疗后获得完全缓解，鉴于患者为青年男性，Ki-67（70％+）且无非血缘HLA相合供者以及血缘供者行allo-HSCT，患者充分知情同意及医院伦理审批（XJTU1AF2025LSYY-922）通过后，探索性进行auto-HSCT序贯CAR-T细胞治疗。

2025年7月29日采集外周血淋巴细胞，并采用靶向CD19的二代CAR结构（含4-1BB共刺激域）的慢病毒载体转导制备CAR-T细胞。给予G-CSF联合普乐沙福稳态动员后采集自体外周血干细胞，8月4日、5日共采集单个核细胞4.98×10^8^/kg，CD34^+^细胞计数6.93×10^6^/kg，采集后给予奥加伊妥珠单抗1 mg治疗1次，整个治疗周期内行鞘内注射3次。2025年9月9日复查PET-CT提示：原左侧后纵隔^18^F-FDG代谢增高灶较前明显好转，相应部位未见异常^18^F-FDG代谢增高灶；新增左侧腋前、下腹壁、双侧大腿根部软组织增厚，^18^F-FDG代谢增高，考虑炎症性病变可能。双侧腋窝、腹股沟反应性淋巴结增生。左侧腋前皮肤软组织活检提示皮肤及皮下纤维组织慢性炎症。采用TEAM方案（塞替派10 mg/kg，−7 d；阿糖胞苷200 mg/m^2^间隔12 h 1次，−6～−3 d；依托泊苷200 mg/m^2^，−6～−3 d；美法仑140 mg/m^2^，−2 d），2025年9月22日（0 d）回输采集的自体外周血造血干细胞。CAR-T细胞产品（纳基奥仑赛注射液，含CAR-T细胞计数0.9×10^8^个），放行前进行质量控制，检测结果合格，可供临床使用。2025年9月29日（+7 d）回输自体CD19 CAR-T细胞，同时接受左乙拉西坦预防癫痫。CAR-T细胞回输后第14天达扩增高峰，之后逐渐下降，扩增的CAR-T细胞主要为CD8^+^ CAR-T细胞。+10 d粒系重建，随着CAR-T细胞扩增，粒系细胞水平再次降低，给予地塞米松治疗后粒系稳定重建，+29 d血小板重建。患者在CAR-T细胞输注后第3天反复发热，最高体温39.2 °C，伴有低血压（87/54 mmHg，1 mmHg＝0.133 kPa），血氧饱和度正常，IL-6水平升高，诊断为2级CRS，给予地塞米松10 mg每6 h 1次治疗后迅速控制。截至2025年12月1日，患者处于MRD阴性缓解状态。

